# A novel early diagnostic framework for chronic diseases with class imbalance

**DOI:** 10.1038/s41598-022-12574-x

**Published:** 2022-05-21

**Authors:** Xiaohan Yuan, Shuyu Chen, Chuan Sun, Lu Yuwen

**Affiliations:** School of Big Data and Software Engineering, Chongqing University, Chongqing, China

**Keywords:** Computational biology and bioinformatics, Health care, Engineering, Mathematics and computing

## Abstract

Chronic diseases are one of the most severe health issues in the world, due to their terrible clinical presentations such as long onset cycle, insidious symptoms, and various complications. Recently, machine learning has become a promising technique to assist the early diagnosis of chronic diseases. However, existing works ignore the problems of feature hiding and imbalanced class distribution in chronic disease datasets. In this paper, we present a universal and efficient diagnostic framework to alleviate the above two problems for diagnosing chronic diseases timely and accurately. Specifically, we first propose a network-limited polynomial neural network (NLPNN) algorithm to efficiently capture *high-level* features hidden in chronic disease datasets, which is data augmentation in terms of its feature space and can also avoid over-fitting. Then, to alleviate the class imbalance problem, we further propose an attention-empowered NLPNN algorithm to improve the diagnostic accuracy for sick cases, which is also data augmentation in terms of its sample space. We evaluate the proposed framework on nine public and two real chronic disease datasets (partly with class imbalance). Extensive experiment results demonstrate that the proposed diagnostic algorithms outperform state-of-the-art machine learning algorithms, and can achieve superior performances in terms of accuracy, recall, F1, and G_mean. The proposed framework can help to diagnose chronic diseases timely and accurately at an early stage.

## Introduction

Chronic diseases have been a severe health issue in the world. In 2019, the World Health Organization pointed out that chronic diseases account for about 7 of the top 10 causes of death in the world^2^. Deaths caused by chronic diseases account for more than 63% of the total global deaths. Common chronic diseases include heart disease, diabetes, hypertension, etc., which are mainly caused by individual unhealthy lifestyles^3^. Once people suffer from chronic diseases, several vital organs (e.g., eye, brain, heart, kidney, etc.) will be damaged, and it is easy to cause a series of serious complications affecting work and life^4^. Patients with chronic diseases are particularly vulnerable to infectious diseases, such as the coronavirus disease 2019 (COVID-19)^5^. More than 48% of COVID-19 patients have a history of chronic diseases and are more likely to develop severe symptoms^6,7^. Additionally, chronic diseases will lead to expensive medical expenses^8^. The Centers for Disease Control and Prevention reports chronic diseases are leading drivers of the nation’s 3.8 trillion in annual health care costs^9^. The main reason for the high fatality rate and expensive medical expenses is that chronic diseases have some terrible clinical presentations such as a long onset cycle, insidious symptoms, irreversible development, and various complications^10^. The above information reminds us that we need to quickly strengthen the prevention, diagnosis, and treatment of chronic diseases. Therefore, the early diagnosis of chronic diseases is urgent and essential, which can motivate high-risk patients to change their unhealthy lifestyles, thereby reducing the incidence of complications and further improving their health and quality of life.

Since the onset of chronic diseases is imperceptible and there are no obvious clinical symptoms in the early stage, it is difficult for doctors to determine the risk of patients with chronic diseases. Nowadays, machine learning has become the hottest promising technology for the assisted diagnosis of diseases with its advantages of autonomous learning and low error rate^11–13^. Several state-of-the-art machine learning algorithms have been widely used in the early diagnosis of different chronic diseases (e.g., chronic kidney disease, diabetes), such as support vector machines (SVM)^14^, logistic regression (LR)^15^, k-nearest neighbor (KNN)^16^, decision trees (DT)^17^, and the ensemble of some algorithms^18–20^. However, existing works are mainly dedicated to data preprocessing (e.g., data regularization and feature selection) to improve the early diagnostic performance of only a certain chronic disease^21,22^. Besides, they ignore the problems of feature hiding and imbalanced class distribution in chronic disease datasets. Hence, these methods are not conducive to improving the performance of the diagnostic model and are not suitable for a universal and efficient diagnosis of chronic diseases.

The problem of feature hiding represents that the feature in the dataset maybe not be directly related to decision-making. It needs to be further comprehensively analyzed together with other features to obtain the features directly related to decision-making^23^. For example, based on the heart rate and body mass index in the data, it is not possible to directly decide whether a patient has heart disease. If the visible original features of the data are directly used, neither the doctor nor the machine learning may be able to make a wise decision. Therefore, we need to expand the feature space of the data to capture its potential features related to chronic disease diagnosis. Additionally, the imbalanced class distribution of the dataset refers to a significant skew that exists between the number of samples for the different classes, which is also called the class imbalance problem^24^. The dominant class is called the majority class, and the remaining classes are called the minority class. Learning from the dataset with the class imbalance problem will make the learned model unreliable, which is more concerned with identifying the majority class correctly and ignoring the minority class^25,26^. Especially, in the chronic disease dataset, the number of sick cases (minority class) is generally lower than the number of healthy cases (majority class). However, the cost of misdiagnosing a sick case as a healthy case is significantly higher than the cost of misdiagnosing a healthy case as a sick case. The former may cause the patient to miss the best treatment period^27^. Therefore, how to accurately identify sick cases from the class imbalanced chronic disease dataset without affecting the overall diagnostic performance is of crucial importance and also a very challenging task.

Deep neural networks have great potential for solving various engineering problems in many fields, by extracting *high-level* features from data to achieve superior classification performance^28,29^. However, most deep neural network algorithms are not friendly to small-scale datasets and are prone to data overfitting^30,31^. Additionally, as the collected chronic disease data are not generally abundant (i.e., small-scale datasets), some existing deep neural network algorithms cannot train a well diagnostic model for chronic diseases. Recently, the deep polynomial neural network (PNN) has received the attention of some researchers^32–34^. We investigate the advantage of PNN and find that PNN is very friendly to classification tasks on small-scale datasets compared to other deep neural network algorithms. Surprisingly, the ideal PNN is parameter-free and can reduce the training error to zero iteratively^35^. Each network node of PNN is a polynomial function of its input. Thus, PNN can represent any polynomial value over the input data. Particularly, similar to other deep neural network algorithms, the network architecture of PNN is constructed layer by layer, which can represent higher and higher level (hidden) features of the input data. In other words, PNN can hierarchically expand the feature space of its input, and effectively capture features related to chronic disease diagnosis. Finally, the output layer of PNN can be constructed by solving a simple convex optimization problem.

In this paper, we are motivated to investigate the issue of the early diagnosis of chronic diseases. To the best of our knowledge, we are the first to study a universal and efficient diagnostic framework for chronic diseases, which can extract *high-level* features and solve the class imbalance problem to diagnose chronic diseases timely and accurately. Specifically, to efficiently capture *high-level* features hidden in chronic disease datasets, we propose a network-limited PNN (NLPNN) algorithm to avoid the problem of over-fitting. NLPNN can be seen as data augmentation in terms of its feature space. Additionally, as collected chronic disease datasets generally have a serious class imbalance problem, that is, the number of positive samples (sick cases) is significantly less than the number of negative samples (healthy cases), the PNN-based diagnostic model cannot fully learn the knowledge of sick cases, resulting in costly misdiagnosis (low recall). To alleviate this class imbalance problem, we further consider empowering samples with attention (i.e., weight) to change the importance of each sample and propose an improved NLPNN algorithm, named attention-empowered NLPNN (AEPNN). AEPNN pays more attention to these samples that are misclassified by NLPNN, regarded as data augmentation in terms of its sample space. Thus, the main contributions of this paper are summarized as follows.

- We study a universal and efficient diagnostic framework to make timely and accurate early diagnosis of chronic diseases with small-scale datasets.
- We propose an NLPNN algorithm to avoid the problem of over-fitting, which can efficiently capture *high-level* features hidden in chronic disease datasets and achieve high classification accuracy.
- We further propose an AEPNN algorithm to solve the class imbalance problem, which greatly improves the recall of the diagnostic model, that is, it can accurately diagnose the sick case.
- We evaluate and compare the proposed methods against other state-of-the-art methods using nine chronic diseases datasets (partly with class imbalance) and extensive experimental results demonstrate that the proposed two diagnostic models outperform state-of-the-art machine learning algorithms, and can achieve superior accuracy and recall.

The rest of the paper is organized as follows. We discuss related work in “Related work” section. “Diagnostic framework for chronic diseases” section presents the proposed algorithms, and experiment results are shown in “Experimental results” section. Finally, “Conclusion” section concludes this paper.

## Related work

### Early diagnosis of chronic diseases

Several existing machine learning algorithms have been proposed to diagnose a certain chronic disease^36–38^. Heydari et al.^36^ compared the performance of various machine learning classification algorithms in the early diagnosis of type 2 diabetes. The simulation results showed that the performance of classification techniques depends on the nature and complexity of the dataset. Khan et al.^37^ developed a chronic disease risk prediction framework. To reduce the impact of outliers, Alirezaei et al.^38^ incorporated K-means clustering, SVM, and meta-heuristic algorithm to diagnose diabetes disease. However, they ignored the influence of data distribution and structural changes on model generalization performance. Under the premise of not changing the structure and distribution of data, the authors in^13^ proposed a diagnostic model based on XGBoost for chronic kidney disease (CKD). Sekar et al.^39^ used a hierarchical neural network fusion method (FHNN) for the stratified diagnosis of cardiovascular disease (CVD). However, the impact of FHNN mainly depends on the optimal choice of the sub-neural network. Some tree-based ensemble learning techniques applied to early diagnosis methods of diabetes were comprehensively studied by Tama et al.^20^, and the differential performance of different classification methods was evaluated through statistical significance tests. At the same time, Altan et al.^40^ also compared various machine learning algorithms for the early diagnosis of chronic obstructive pulmonary disease and proposed a deep learning model to analyze multi-channel lung sounds using statistical features of Hilbert-Huang transform, which successfully achieved high classification performance of accuracy, sensitivity, and specificity of 93.67%, 91%, and 96.33%, respectively.

### Class imbalance

In medical datasets, the problem of class imbalance seriously affects the accuracy of classifiers^27,24^. In most cases, it directly leads to a high rate of misdiagnosis of the disease. This is because the class imbalance of the training data brings difficulties to the algorithm learning, and the algorithm pays more attention to the majority class^41^. However, the minority class in medical datasets (sick vs. healthy) is often more important from a data mining perspective, and it usually carries critical and useful knowledge. At present, many scholars have studied the class imbalance problem, among which there are three main methods to alleviate the class imbalance^42,43^. (1) Data-level methods: in the data preprocessing stage, re-sampling is used to reduce the size of the majority class or increase the size of the minority class (or both) to balance the training set and eliminate difference. (2) Algorithm-level methods: in the training phase, the learning algorithm is modified to be suitable for mining data with imbalanced distributions. (3) Hybrid methods: the advantages of the first two methods are combined to alleviate the adverse effects of class imbalance on the results.

## Diagnostic framework for chronic diseases

Statement: I confirm that all methods were performed in accordance with the relevant guidelines and regulations.

In this section, we propose a universal and efficient diagnostic framework for diagnosing chronic diseases timely and accurately. The proposed framework consists of the NLPNN algorithm and AEPNN algorithm to alleviate the problems of feature hiding and class imbalance, respectively.

### Network-limited polynomial neural network

**Figure 1:**
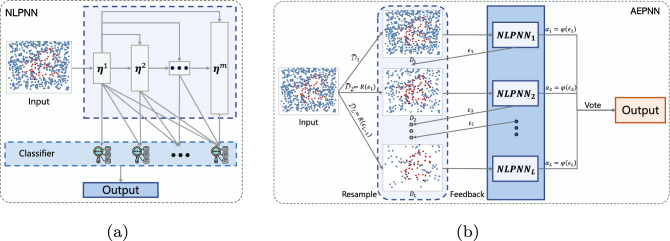
Flowchart of the proposed algorithms: (**a**) NLPNN; (**b**) AEPNN.

The PNN algorithm is dedicated to learning the high-level polynomial feature representation of the data through multi-layer network architecture, and finally, output features hierarchically^32,33^. Although the PNN algorithm has been proven to run in polynomial time, it still has a limitation, that is, the depth and width of the network cannot be controlled. Its network depth and width are both adaptive, and the criterion for depth stopping is until the training error is zero^35^. In the worst case, the network depth can be infinitely deepened or the network width can be as large as the number of training samples *n*. This will lead to severe overfitting. Hence, we present an NLPNN algorithm for the early diagnosis of chronic diseases to avoid this issue. The structure of NLPNN is shown in Fig. 1a, and the details of the NLPNN algorithm applied to chronic diseases diagnosis be described below.

For the early diagnosis of chronic diseases, we denote the labeled training dataset as ${\varvec{D}}= ({\varvec{X}},{\varvec{y}})$, where ${\varvec{X}} \in {\mathbb {R}} ^{n\times d}$ is the set of *n* samples with *d* features; ${\varvec{y}}=\left( y_{1},y_{2},\ldots ,y_{n}\right) ^{T}$ is a *n*-dimensional column vector and $y_{i}\in \left\{ -1,1\right\}$, $\forall i=1,2,\ldots ,n$. Here, $y_{i}$ = 1 means that the *i*-th sample is labeled as a sick case, and $y_{i}$ = − 1 otherwise. The *M*-order multivariate polynomial on the sample ${\varvec{x}}_{i}=\left( x_{i1},\ldots ,x_{id}\right) \in {\varvec{X}}$ is written as

1 $$\begin{aligned} {\varvec{p}}({\varvec{x}}_{i})=\sum _{j=0}^{M} \sum _{\varvec{\alpha }^{(j)}} w_{\varvec{\alpha }^{(j)}} \prod _{s=1}^{d} x_{is}^{\alpha _{s}^{(j)}}, \end{aligned}$$

where $\varvec{\alpha }^{(j)}$ is a *d*-dimensional vector composed of non-negative integers and $\sum _{s=1}^{d} \alpha _{s}^{(j)}\!=\!j$; $w_{\varvec{\alpha }^{(j)}}$ is a coefficient of monomial $\prod _{s=1}^{d} x_{is}^{\alpha _{s}^{(j)}}$ of degree *j*. Represent the value of each polynomial ${\varvec{p}}$ on *n* samples by linear projection

2 $$\begin{aligned} {\varvec{p}} \mapsto \left( {\varvec{p}}\left( {\varvec{x}}_{1}\right) , \ldots , {\varvec{p}}\left( {\varvec{x}}_{n}\right) \right) ^T. \end{aligned}$$

According to linear algebra, there are *n* polynomials ${\varvec{p}}_{1},\ldots ,{\varvec{p}}_{n}$, and $\left\{ \left( {\varvec{p}}_{i}\left( {\varvec{x}}_{1}\right) , \ldots , {\varvec{p}}_{i}\left( {\varvec{x}}_{n}\right) \right) ^T\right\} _{i=1}^{n}$ form a basis of ${\mathbb {R}} ^{n}$ space. Therefore, there is a coefficient vector $\varvec{\nu } = (\nu _1,\ldots ,\nu _n)$, so that $\sum _{i=1}^{n} \nu _{i} {\varvec{p}}_{i}\left( {\varvec{x}}_{j}\right) =y_{j}, \forall y_{j} \in (y_1,\ldots ,y_n)^T\in {\mathbb {R}} ^{n}$.

The network layer of PNN is constructed by solving the basis of polynomial hierarchically, and each node calculates a linear function or weighted product over its input. We denote the *j*-th node of the *i*-th layer as $\eta _j^i(\cdot )$, which actually represents a feature (original or high-level) of the input data. For the first layer, the *j*-th node is the degree-1 polynomial (or linear) function $\eta _{j}^{1}({\varvec{x}})=[1 \ {\varvec{x}}]{\varvec{w}}_{j}$, and the $\left\{ \left( \eta _{j}^{1}\left( {\varvec{x}}_{1}\right) , \ldots , \eta _{j}^{1}\left( {\varvec{x}}_{n}\right) \right) ^{T}\right\} _{j=1}^{d+1}$ is the basis of all values obtained by a polynomial of degree 1 on the training dataset. They form the columns of matrix $F^1\in {{\mathbb {R}}^{n\times (d+1)}}$ and $F_{i, j}^{1}=\eta _{j}^{1}\left( {\varvec{x}}_{i}\right)$. So far, a single-layer network has been constructed, and its output spans all the values obtained by the linear function on the training sample.

Generally speaking, the basis of the degree-2,3,...*M* polynomial is also obtained in the same trick. However, we find that the basis of the degree-*M* multiple polynomials is composed of $(d+1)^M$ vector elements. The scale of the basis of the polynomial increases exponentially with its degree, which will run into a computational problem.

The work in^35^ indicates that any degree-*m* polynomial can be regraded as

3 $$\begin{aligned} \sum _{i}{\varvec{g}}_{i}( {\varvec{x}}) {\varvec{h}}_{i}( {\varvec{x}})+{\varvec{k}}( {\varvec{x}}), \end{aligned}$$

where ${\varvec{g}}_{i}({\varvec{x}})$ and ${\varvec{h}}_{i}( {\varvec{x}})$ are degree-1 and degree-$(m-1)$ polynomials respectively; ${\varvec{k}}( {\varvec{x}})$ is a polynomial of degree not greater than $m-1$. Since all degree-1 polynomials are spanned by the nodes at the first layer of PNN, any degree-2 polynomial can be written as

4 $$\begin{aligned} \sum _{i}\left( \sum _{j} \alpha _{j}^{\left( {\varvec{g}}_{i}\right) } \eta _{j}^{1}({\varvec{x}})\right) \left( \sum _{r} \alpha _{r}^{\left( {\varvec{h}}_{i}\right) } \eta _{r}^{1}({\varvec{x}})\right) +\left( \sum _{j} \alpha _{j}^{({\varvec{k}})} \eta _{j}^{1}({\varvec{x}})\right) , \end{aligned}$$

where $\alpha _{j}^{\left( {\varvec{g}}_{i}\right) }$, $\alpha _{r}^{\left( {\varvec{h}}_{i}\right) }$, $\alpha _{j}^{({\varvec{k}})}$ are scalar multipliers. (4) implies that the construction of the second layer of the network is based on the first layer. The matrix $[F^{1} {\tilde{F}}^{2}]$ is formed by concatenating the columns of $F^{1}$, ${\tilde{F}}^{2}$, which spans all values attainable by degree-2 polynomials, and

5 $$\begin{aligned} {\tilde{F}}^{2}=[\left( F_{1}^{1} \circ F_{1}^{1}\right) \cdots \left( F_{1}^{1} \circ F_{\left| F^{1}\right| }^{1}\right) \cdots \left( F_{\left| F^{1}\right| }^{1} \circ F_{1}^{1}\right) \cdots \left( F_{\left| F^{1}\right| }^{1} \circ F_{\left| F^{1}\right| }^{1}\right) ], \end{aligned}$$

where the symbol $\circ$ indicates the Hadamard product; $F_{1}$ refers to the first column of *F*; $\left| F\right|$ refers to the number of columns of *F*. Similar to degree-1 polynomial, the column subset $F^{2}$ of ${\tilde{F}}^{2}$ should be found, so that the column of $[F^{1} F^{2}]$ are the basis of column of $[F^{1} {\tilde{F}}^{2}]$. The second layer of the PNN is constructed by the column of $F^{2}$, which is the product of two nodes $\eta _{i}^{1}(\cdot )$ and $\eta _{j}^{1}(\cdot )$ in the first layer.

The next step is to repeat the above process. Successively, the $m = 3, 4, \ldots , M$ layers of the network are constructed. We represent the matrix, written as

6 $$\begin{aligned} {\tilde{F}}^{m}=[\left( F_{1}^{m-1} \circ F_{1}^{1}\right) \cdots \left( F_{1}^{m-1} \circ F_{\left| F^{1}\right| }^{1}\right) \cdots \left( F_{\left| F^{m-1}\right| }^{m-1} \circ F_{1}^{1}\right) \cdots \left( F_{\left| F^{m-1}\right| }^{m-1} \circ F_{\left| F^{1}\right| }^{1}\right) ]. \end{aligned}$$

Thus, we find a linearly independent column subset $F^{m}$ of ${\tilde{F}}^{m}$, which lets the columns of matrix $[F \ F^{m}]$ are a basis of the columns of the augmented matrix $[F \ {\tilde{F}}^{m}]$, where the columns of $F=\left[ F^{1}\ F^{2}\ \ldots \ F^{m-1}\right]$ can span the values attained by all polynomials for degree at most $m-1$ over the training dataset. In addition, it needs to be explained that the conversion of ${\tilde{F}}^{m}$ to $F^{m}$ is achieved by

7 $$\begin{aligned} F_{s}^{m}:=W_{i(s), j(s)} F_{i(s)}^{m-1} \circ F_{j(s)}^{1},s=1,\ldots ,|F^{m}| , \end{aligned}$$

where the projection matrix $W\in {\mathbb {R}}^{{|F^{m-1}|\times |F^{1}|}}$ and $W_{i(s), j(s)}=\sqrt{n} /\left\| {\tilde{F}}_{s}^{m}\right\|$. Therefore, when the *M*-layer network of the PNN is constructed, all the values obtained by the polynomial of degree at most *M* over the training dataset can be spanned by the columns of the matrix *F*. In fact, *F* stores the high-level features of the input data, the deeper layer, the higher feature.

However, for the implementation of NLPNN, we use a parameter $\Omega =(d+1,\cdots ,d+1) \in {\mathbb {Z}}^M$ to pre-limit the depth and width of the network, which represents that the network consists of *M* ($|\Omega |$) non-output layers and each layer has $d+1$ nodes at most. In the first non-output layer, we use singular value decomposition on the augmented data matrix $[{\varvec{1}}\ {\varvec{X}}]$ to obtain its partial orthogonal basis, which forms the $d+1$ nodes (select the first $d+1$ main singular vectors). In the next non-output layer, a standard Orthogonal Least Squares (OLS) procedure is utilized to greedily select the partial orthogonal basis which are the first $d+1$ relevant features for diagnosis of chronic disease according to the established high-level feature set ${\tilde{F}}^{m}$. Finally, a simple linear classifier $\varvec{\nu }_{m}$ with input data $F=\left[ F^{1}\ F^{2}\ \ldots \ F^{m}\right]$ is trained. Therefore, there are *M* linear classifiers in the output layer. It should be pointed out that each linear classifier $\varvec{\nu }_{m}$ is trained by a stochastic gradient descent method, which is utilized to solve the $L_{2}$ regularization problem

8 $$\begin{aligned} \min _{\varvec{\nu }_{m},\lambda _{m}}\frac{1}{n} \sum _{i=1}^{n} \ell _{i}\left( [F^{1}\ \cdots \ F^{m}]_{i\cdot }\cdot \varvec{\nu }_{m},y_{i}\right) +\lambda _{m}\Vert \varvec{\nu }_{m}\Vert _{2}, \end{aligned}$$

where $\ell _{i}\left( F^{m}_{i\cdot }\cdot \varvec{\nu },y_{i}\right) =\max (0,1-(F^{m}_{i\cdot }\cdot \varvec{\nu })\cdot y_{i})$ is a hinge loss and $F^{m}_{i\cdot }$ represents the *i*-th row of matrix $F^{m}$; $\lambda _{m} \in \Lambda$ is the regularization factor. Then combined with the value set $\Lambda$ of the regularization factor, we check the network performance layer by layer on the verification dataset to find the optimal network layer and the best regularization factor. Finally, an optimal linear classifier $\varvec{\nu }^{*}$ is obtained by

9 $$\begin{aligned} \min _{m}\min _{\varvec{\nu }_{m},\lambda _{m}}\frac{1}{n} \sum _{i=1}^{n} \ell _{i}\left( [F^{1}\cdots F^{m}]_{i\cdot }\cdot \varvec{\nu }_{m},y_{i}\right) +\lambda _{m}\Vert \varvec{\nu }_{m}\Vert _{2}, \end{aligned}$$

and the output is this optimal classifier. The purpose of NLPNN is to adaptively find features related to diagnosis from the augmented data that is augmented in terms of its feature space. The detailed process of NLPNN is shown in Algorithm 1, which briefly describes the entire process from the establishment of the network layer to the acquisition of the output layer.
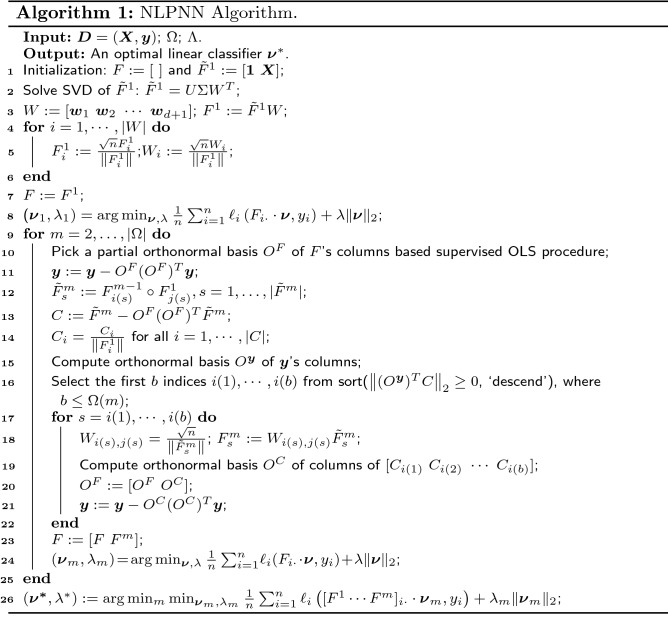


### Attention-empowered NLPNN

Some chronic disease datasets exist the class imbalance problem, where sick cases are generally scarce compared to healthy cases. However, the correct diagnosis of the minority sick cases among all cases is vital in a healthcare system. The reason is that the cost of misdiagnosing sick cases is much higher than healthy cases, where the latter only requires further examination and the former carries a life-threatening risk. During the training phase of NLPNN, since the samples of each class in the imbalanced dataset are utilized equally, the trained model tends to bias towards the majority class and ignore the samples (sick cases) in the minority class. Thus, NLPNN does not perform well in dealing with class imbalance problems and causes serious misdiagnosis of minority sick cases. Furthermore, for the early diagnosis of chronic diseases, although we are more concerned with the accurate diagnosis of sick cases, we cannot ignore the overall diagnostic accuracy. To alleviate the class imbalance problem, we empower the cases with attention (i.e., weight) and propose an AEPNN algorithm. AEPNN pays more attention to the cases misdiagnosed by NLPNN by changing the importance of these cases. Motivated by committee-based learning^25^, AEPNN trains and combines multiple complementary NLPNN to further improve the performance of NLPNN in alleviating the class imbalance problem. The structure of AEPNN is shown in Fig. 1b.

For the implementation of AEPNN, we first assign an identical initial weight ${\mathcal {D}}_{1}({\varvec{x}})=\frac{1}{n}$ to each sample ${\varvec{x}}$ in the training dataset. An NLPNN classifier $h_{1}$ is trained from the training dataset ${\varvec{D}}_1$ with the initialized weight distribution ${\mathcal {D}}_{1}$ and $h_{1}$’s error $\epsilon _{1}$ is fed back to the training sample, so that the training sample’s distribution is adjusted by ${\mathcal {D}}_{2}({\varvec{x}})$. Then, the second NLPNN classifier $h_{2}$ is trained from the training dataset ${\varvec{D}}_2$ with the weight distribution ${\mathcal {D}}_{2}$, where the weights of samples misdiagnosed by $h_{1}$ are increased in ${\mathcal {D}}_{2}$ to make $h_{2}$ pay more attention to the samples that are misdiagnosed by $h_{1}$. This process is repeated until $h_{L}$ is trained after *L* iterations. Finally, the predicted label is obtained through the weighted combination of all NLPNN classifiers. The main process is shown in Algorithm 2.
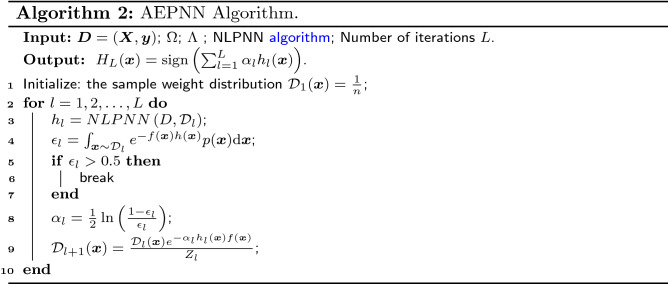


Specifically, we denote the true label corresponding to sample ${\varvec{x}}$ as $f({\varvec{x}})$, and the predicted label obtained by the NLPNN classifier as $h({\varvec{x}})$. Obviously, the loss function $\epsilon$ is defined as

10 $$\begin{aligned} \epsilon =\int _{{\varvec{x}} \sim {\mathcal {D}}} {\mathbb {I}}\left( f({\varvec{x}}) \ne h({\varvec{x}})\right) p({\varvec{x}}) {\mathrm {d}} {\varvec{x}}, \end{aligned}$$

where $p({\varvec{x}})$ represents the probability density function of ${\varvec{x}}$ following the data distribution ${\mathcal {D}}$. However, $\epsilon$ has poor mathematical properties (non-convex and non-continuous), which makes it very difficult to be solved directly. To optimize the loss function more conveniently, we select a convex and continuously differentiable exponential loss function (11) to replace the loss function (10). Lemma 1 proves that $\ell _{\exp }(h \mid {\mathcal {D}})$ is the consistent replacement of the loss function $\epsilon$, which means that (11) can replace (10) to update the weight ${\mathcal {D}}_{l}({\varvec{x}})$ of the sample and the weight $\alpha _{l}$ of the classifier in Algorithm 2.

#### **Lemma 1**

*The consistent replacement of the loss function* $\epsilon$ *is the exponential loss function*

11 $$\begin{aligned} \ell _{\exp }(h \mid {\mathcal {D}})=\int _{{\varvec{x}} \sim {\mathcal {D}}} e^{-f({\varvec{x}}) h({\varvec{x}})} p({\varvec{x}}) {\mathrm {d}} {\varvec{x}}. \end{aligned}$$

#### *Proof*

Please see Appendix 1. $\square$

In Algorithm 2, the $h_1$ is obtained by applying the NLPNN classifier to the initial samples distribution ${\mathcal {D}}_{1}$. When $h_{l}$ is generated based on distribution ${\mathcal {D}}_{l}$, the weight $\alpha _{l}$ of the classifier $h_{l}$ is obtained iteratively by minimize the exponential loss function $\ell _{\exp }\left( \alpha _{l} h_{l} \mid {\mathcal {D}}_{l}\right)$. From Lemma 2, we know that $\alpha _{l}=\frac{1}{2} \ln \left( \frac{1-\epsilon _{l}}{\epsilon _{l}}\right)$ is a necessary and sufficient condition for the exponential loss function $\ell _{\exp }\left( \alpha _{l} h_{l} \mid {\mathcal {D}}_{l}\right)$ to obtain the minimum value. It means that, under the encouragement of the weight $\alpha _{l}$, the classifier $h_{l}$ can achieve the best performance on the dataset ${\varvec{D}}_l$ with distribution ${\mathcal {D}}_{l}$.

#### **Lemma 2**

*The exponential loss function* $\ell _{\exp }\left( \alpha _{l} h_{l} \mid {\mathcal {D}}_{l}\right)$ *at* $\alpha ^{*}_{l}h_{l}$ *obtain the minimum value*, *where* $\alpha ^{*}_{l}=\frac{1}{2} \ln \left( \frac{1-\epsilon _{l}}{\epsilon _{l}}\right)$ *and* $\epsilon _{l}=\int _{{\varvec{x}} \sim {\mathcal {D}}_{l}} {\mathbb {I}}(f({\varvec{x}}) \ne h_{l}({\varvec{x}})) p({\varvec{x}}) {\mathrm {d}} {\varvec{x}}$.

#### *Proof*

Please see Appendix 2. $\square$

$H_{l}$ is the voting result of the first *l* NLPNN classifier $\{h_{i}\}_{1}^{l}$ with weights $\{\alpha _{i}\}_{1}^{l}$, and its error can be corrected by the next classifier $h_{l+1}$. Ideally, $h_{l+1}$ can correct all errors of $H_{l}$ by minimizing the exponential loss $\ell _{\exp }(H_{l}+h \mid {\mathcal {D}})$. From Lemma 3, all errors of $H_{l}$ can be corrected by the NLPNN classifier $h_{l+1}$ which is trained based on the sample weight distribution ${\mathcal {D}}_{l+1}({\varvec{x}})={{\mathcal {D}}_{l}({\varvec{x}}) \frac{e^{-\alpha _{l}f({\varvec{x}}) h_{l}({\varvec{x}})}}{Z_{l}}}$, where $\frac{1}{Z_{l}}=\frac{\int _{{\varvec{x}} \sim {\mathcal {D}}}\left[ e^{- f({\varvec{x}}) H_{l-1}({\varvec{x}})}\right] p({\varvec{x}}) {\mathrm {d}} {\varvec{x}}}{\int _{{\varvec{x}} \sim {\mathcal {D}}}\left[ e^{- f({\varvec{x}}) H_{l}({\varvec{x}})}\right] p({\varvec{x}}) {\mathrm {d}} {\varvec{x}}}$ is the normalization factor to ensure that ${\mathcal {D}}_{l+1}$ is a distribution.

#### **Lemma 3**

*Assume that the base classifier* $h_{1}$ *is generated based on the data distribution* ${\mathcal {D}}_{1}$, *and* $\alpha _{i}$ *is the weight of the classifier* $h_{i}$, $H_{l}({\varvec{x}})={\text {sign}}\left( \sum _{i=1}^{l} \alpha _{i} h_{i}({\varvec{x}})\right)$, $l=1,2,\ldots$, *then all the false predictions of* $H_{l}$ *can be corrected through the ideal base classifier* $h_{l+1}$, *which is generated based on the data distribution*

12 $$\begin{aligned} \begin{aligned} {\mathcal {D}}_{l+1}({\varvec{x}})={{\mathcal {D}}_{l}({\varvec{x}}) e^{-f({\varvec{x}}) \alpha _{l} h_{l}({\varvec{x}})}} \frac{\int _{{\varvec{x}} \sim {\mathcal {D}}}\!e^{- f({\varvec{x}}) H_{l-1}({\varvec{x}})}\!p({\varvec{x}}) {\mathrm {d}} {\varvec{x}}}{\int _{{\varvec{x}} \sim {\mathcal {D}}}\!e^{- f({\varvec{x}}) H_{l}({\varvec{x}})}\!p({\varvec{x}}) {\mathrm {d}} {\varvec{x}}}. \end{aligned} \end{aligned}$$

#### *Proof*

Please see Appendix 3. $\square$

In summary, we iteratively optimize the exponential loss function by introducing two kinds of attention (${\mathcal {D}}$ and $\alpha$) to achieve the superiority of AEPNN on class-imbalanced datasets.

## Experimental results

Some DNN models are not suitable for classification tasks with the small-scale dataset due to the over-fitting problem. However, the PNN-based deep learning algorithm performs well for the early diagnosis of chronic diseases with the small-scale dataset, due to its unique network structure. We select five state-of-the-art machine learning algorithms as the baseline algorithms, i.e. SVM^44^, LR^45^, KNN^46^, DT^47^, and multi-layer perceptron (MLP)^48^.

### Chornic disease datasets

To verify the effectiveness of the proposed algorithm in the early diagnosis of chronic diseases, we select nine public and two private chronic disease datasets for experiments. Nine public chronic disease datasets (i.e., http://archive.ics.uci.edu/ml, https://www.kaggle.com/datasets) include CKD, Pima Indian diabetes dataset (PIMA), CVD, Heart Disease Dataset (Heart), Framingham Heart Disease dataset (Fra_Heart), Hepatitis dataset (Hep), Breast Cancer Wisconsin dataset (BCW) in UCI Machine Learning Repository, Type 2 Diabetes Mellitus Dataset (T2DM) and Gestational Diabetes Mellitus dataset (GDM) in the Tianchi Precision Medicine Competition. They are scarce and precious, but some of them have problems, such as small size, class imbalance, and missing value. Two private chronic disease datasets (Pri_hyper dataset and Pri_diab dataset) are collected from a district in Chongqing, China. The Pri_hyper dataset consists of the health records of hypertensive patients and healthy people. The Pri_diab dataset consists of the health records of diabetic patients and healthy people. The composition details of the selected datasets are listed by Table 1, in which column *Datasets* is shorthand for the name of the dataset; column *Features* represents the number of features; column *Samples* represents the number of samples; column *Positive: Negative* represents the ratio of the number of positive and negative samples; column *Missing* shows whether there are missing values in the corresponding dataset. Consistently, we split each chronic disease dataset randomly into a training dataset and testing dataset with 8:2, and maintain the distribution of the class before the split. For baseline algorithms that have to process missing values and regularize data, we fill the missing values with zeros and regularize the data. The implementation of proposed algorithms does not require any other data preprocessing technology.

**Table 1:** The composition details of chronic disease datasets.

Datasets	Features	Samples	Positive:Negative	Missing
CKD	24	400	1:0.6	No
PIDD	8	768	1:1.87	No
T2DM	40	5642	1:11.19	Yes
CVD	11	70000	1:1.001	No
Heart	13	1025	1:0.95	No
GDM	83	1000	1:1.13	Yes
Fra_Heart	15	4240	1:5.58	Yes
Hep	19	155	1:0.24	Yes
BCW	10	699	1:1.9	Yes
Pri_hyper	33	9091	1:1.13	No
Pri_diab	28	14,525	1:12.78	No

### Evaluation measurements

For the early diagnosis of chronic diseases, the generalization performance can be estimated on the test dataset. In addition to using the area under the receiver operating characteristic curve (AUC) to evaluate the performance of the model, we also selected the following evaluation indicators to evaluate the proposed algorithm:

- $Accuracy =\frac{TP+TN}{TP+TN+FP+FN}$ represents the ratio of the number of correctly predicted specific classes to the total number of samples.
- $Specificity =\frac{TN}{TN+FP}$ represents the ratio of the number of correctly predicted healthy cases to the total healthy cases.
- $Precision=\frac{TP}{TP+FP}$ represents the ratio of the number of correctly predicted sick cases to the total predicted sick case.
- $Recall=\frac{TP}{TP+FN}$ represents the ratio of the number of correctly predicted sick cases to the total number of sick cases.
- $$F1\_score=\frac{2*Precision*Recall}{Precision+Recall} = \frac{2*TP}{N+TP-TN}$$ is defined based on the harmonic average of precision and recall.

where TP, FP, TN, and FN represent true positive, false positive, true negative and false negative respectively; *N* is the total number of samples.

### Comparison of performance

**Figure 2:**
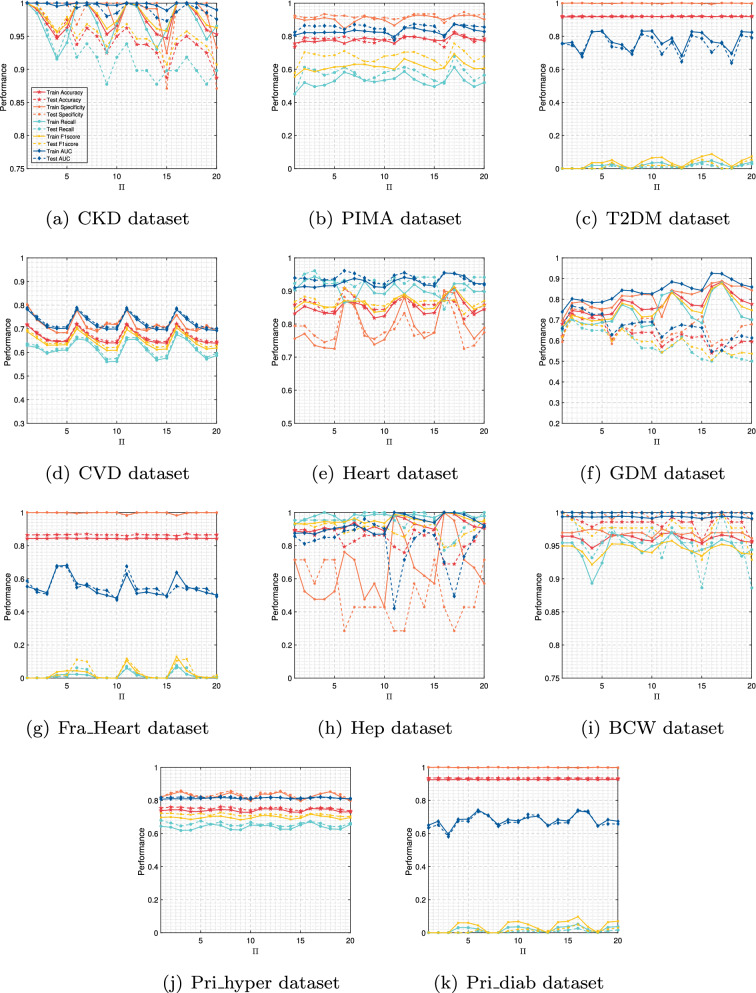
Training and test performance versus $(\Delta ,\lambda )$ on eleven chronic disease datasets.

We investigate the impact of different network depths $\Delta$ and regularization factor $\lambda$ in the NLPNN model for the diagnostic performance of eleven chronic diseases, where $\Delta \in \{2,3,4,5\}$ (network layer plus output layer) and $\lambda \in \Lambda =\{10^{-3},10^{-2}, 10^{-1},10^{0},10^{1}\}$. To visually find the most suitable $\Delta$ and $\lambda$, we combine them into a binary set $(\Delta ,\lambda )$, and establish a bijection function between $(\Delta ,\lambda )$ and $\Pi \in \{1,2,\cdots ,20\}$ described in Table 2. We set $\Pi$ as the horizontal axis to indirectly draw the generalization performance curve of NLPNN with network depth and regularization factor. From Fig. 2, we can see that NLPNN has two advantages in the diagnosis of all chronic diseases, that is, there is no over-fitting phenomenon; the training accuracy is increasing with the increase of the number of network layers (it can be observed that when $\Pi$=1,6, 11,...). However, different $\Pi$ values will affect the performance of the NLPNN algorithm, the impact on different chronic disease datasets is different.

**Table 2:** The bijective relationship between $(\Delta ,\lambda )$ and $\Pi$.

		Regularization factor $(\lambda )$				
		${10^{-3}}$	${10^{-2}}$	${10^{-1}}$	${10^{\ 0}}$	${10^{\ 1}}$
Depth $(\Delta )$	2	1	2	3	4	5
	3	6	7	8	9	10
	4	11	12	13	14	15
	5	16	17	18	19	20

Figure 2a shows that NLPNN can achieve 100% generalization performance on the CKD dataset when $\Pi =\{1,2\}$. Then, with the increase of $\Delta$ and the change of $\lambda$, the test performance decreases somewhat, but both fluctuate within the range of 5%. It means that only a shallow polynomial neural network model can accurately diagnose chronic kidney disease. We can see from Fig. 2b, c, g and k that the $\Pi$ value has almost no effect for the diagnostic accuracy of diabetes and heart disease. In particular, for the diagnosis of hepatitis B disease (Fig. 2h), although the accuracy of the NLPNN model does not vary greatly, its specificity is unstable with the change of $\Pi$ value. This reason is that the Hep dataset has only 155 samples and the negative samples only account for 24% of the total samples. In addition, we can find the best output performance $P^{*}$ of NLPNN and the corresponding value $\Pi ^{*}$ on eleven chronic disease datasets from the Fig. 2. Therefore, according to Table 2, we can find the network structure $\Omega ^{*}$ and the regularization factor $\lambda ^{*}$ when NLPNN achieves the best performance, as shown in Table 3.

**Table 3:** Optimal parameter settings for different datasets.

		Network parameter	
		$\Omega ^{*}$	$\lambda ^{*}$
Dataset	CKD	$$\left[ {\begin{array}{*{20}l} {24} &{} {} &{} {} &{} {} \\ \end{array} } \right]$$	$10^{-3}$
	PIMA	$$\left[ {\begin{array}{*{20}l} {9} &{} {9} &{} {9} &{} {9} \\ \end{array} } \right]$$	$10^{-2}$
	T2DM	$$\left[ {\begin{array}{*{20}l} {32} &{} {32} &{} {32} &{} {} \\ \end{array} } \right]$$	$10^{-3}$
	CVD	$$\left[ {\begin{array}{*{20}l} {12} &{} {12} &{} {12} &{} {12} \\ \end{array} } \right]$$	$10^{-3}$
	Heart	$$\left[ {\begin{array}{*{20}l} {13} &{} {13} &{} {} &{} {} \\ \end{array} } \right]$$	$10^{-2}$
	GDM	$$\left[ {\begin{array}{*{20}l} {84} &{} {} &{} {} &{} {} \\ \end{array} } \right]$$	$10^{-2}$
	Fra_Heart	$$\left[ {\begin{array}{*{20}l} {14} &{} {14} &{} {14} &{} {14} \\ \end{array} } \right]$$	$10^{-2}$
	Hep	$$\left[ {\begin{array}{*{20}l} {14} &{} {14} &{} {14} &{} {14} \\ \end{array} } \right]$$	$10^{\ 0}$
	BCW	$$\left[ {\begin{array}{*{20}l} {11} &{} {11} &{} {11} &{} {11} \\ \end{array} } \right]$$	$10^{-2}$
	Pri_hyper	$$\left[ {\begin{array}{*{20}l} {32} &{} {32} &{} {} &{} {} \\ \end{array} } \right]$$	$10^{-2}$
	Pri_diab	$$\left[ {\begin{array}{*{20}l} {29} &{} {29} &{} {29} &{} {} \\ \end{array} } \right]$$	$10^{-1}$

The generalization performance comparison of baseline algorithms and NLPNN algorithm on eleven chronic disease datasets are shown in Table 4, which lists the test performance results under the unified standard. In general, the diagnostic accuracy of NLPNN on the eleven chronic disease datasets is better than baseline algorithms. Especially for the diagnosis of chronic kidney disease and breast cancer, NLPNN can achieve a generalization accuracy, recall, and F1_score, of 1.0000, 1.0000, and 1.0000, respectively. In addition, NLPNN also shows significant advantages in the diagnosis of Hepatitis disease, and its generalization accuracy is about 10% better than the baseline algorithms (SVM:0.8000, LR: 0.8333, KNN: 0.8000, DT: 0.8333, MLP: 0.8000).

**Table 4:** Performance of different algorithms.

Abbreviation		Acc	Re	F1_score	Abbreviation		Acc	Re	F1_score
CKD	SVM	0.9875	0.9800	0.9899	Fra_Heart	SVM	0.8349	0.0000	0.0000
	LR	0.9875	0.9800	0.9899		LR	0.8420	0.1000	**0.1728**
	KNN	0.9500	0.9200	0.9583		KNN	0.8337	0.0357	0.0662
	DT	0.9750	0.9600	0.9796		DT	0.8361	0.0643	0.1146
	MLP	0.9875	0.9800	0.9899		MLP	0.8314	**0.1357**	0.2099
	**NLPNN**	**1.0000**	**1.0000**	**1.0000**		**NLPNN**	**0.8726**	0.0614	0.1148
PIDD	SVM	0.7792	0.5517	0.6531	Hep	SVM	0.8000	1.0000	0.8846
	LR	0.7987	0.6207	0.6990		LR	0.8333	0.9565	0.8979
	KNN	0.7662	0.4827	0.6086		KNN	0.8000	0.9130	0.8749
	DT	0.7468	0.4310	0.5618		DT	0.8333	1.0000	0.9019
	MLP	0.6559	0.2414	0.3457		MLP	0.8000	0.9130	0.8749
	**NLPNN**	**0.8247**	**0.6774**	**0.7568**		**NLPNN**	**0.9310**	1.0000	**0.9565**
T2DM	SVM	0.9179	0.0000	0.0000	BCW	SVM	0.9714	0.9545	0.9545
	LR	0.9202	**0.0733**	**0.1311**		LR	0.9714	0.9545	0.9545
	KNN	0.9164	0.0092	0.0176		KNN	0.9714	0.9545	0.9545
	DT	0.9187	0.0092	0.0182		DT	0.9500	0.9545	0.9231
	MLP	0.9179	0.0092	0.0180		MLP	0.3143	1.0000	0.4783
	**NLPNN**	**0.9232**	0.0097	0.0192		**NLPNN**	**1.0000**	1.0000	**1.0000**
CVD	SVM	0.7244	0.6401	0.6977	Pri_hyper	SVM	0.7372	0.6162	0.6859
	LR	0.7249	0.6858	0.7125		LR	0.7350	0.6494	0.6953
	KNN	0.6354	0.5389	0.5950		KNN	0.7570	**0.6765**	0.7216
	DT	0.7247	0.6730	0.7085		DT	0.7224	0.4663	0.6100
	MLP	0.5385	**0.9815**	0.6789		MLP	0.7009	0.6210	0.6591
	**NLPNN**	**0.7265**	0.6847	**0.7161**		**NLPNN**	**0.7624**	0.6706	**0.7266**
Heart	SVM	0.8780	0.9307	0.8826	Pri_diab	SVM	0.9280	0.0000	0.0000
	LR	0.8780	0.9109	0.8804		LR	0.9315	0.0478	0.0913
	KNN	0.9024	0.8911	0.9000		KNN	0.9294	**0.1244**	**0.2023**
	DT	0.8488	0.8317	0.8442		DT	0.9325	0.0718	0.1327
	MLP	0.8878	0.8911	0.8866		MLP	0.9322	0.0861	0.1545
	**NLPNN**	**0.9073**	**0.9320**	**0.9100**		**NLPNN**	**0.9360**	0.0053	0.0106
GDM	SVM	0.6500	0.5591	0.5977					
	LR	0.6150	0.5376	0.5649					
	KNN	0.6200	0.4194	0.5064					
	DT	0.6950	0.5269	0.6164					
	MLP	0.6250	0.4839	0.5455					
	**NLPNN**	**0.7300**	**0.7021**	**0.7097**					

The best results for each dataset are marked in bold.

**Figure 3:**
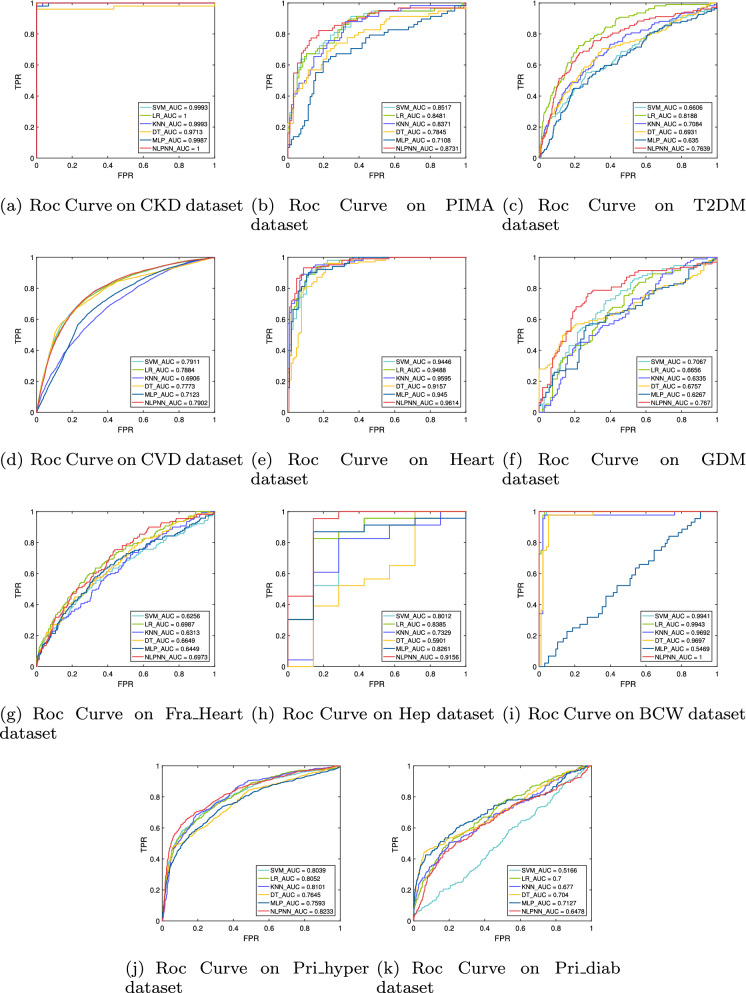
ROC curves of different algorithms with the corresponding AUC values on chronic disease datasets.

Figure 3 plots the ROC curves to further compare the performance of the NLPNN algorithm and the baseline algorithms. The AUC value of the proposed algorithm is generally better than baseline algorithms. It is also worth noting that in the diagnosis task of chronic kidney disease and breast cancer, the NLPNN model is an “ideal model” with an AUC value of 1 (Fig. 3a, i).

**Figure 4:**
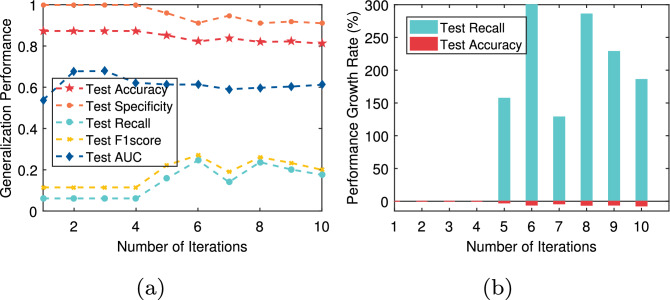
The test performance versus number of iteration on Fra_Heart dataset: (**a**) generalization performance; (**b**) performance growth rate.

**Figure 5:**
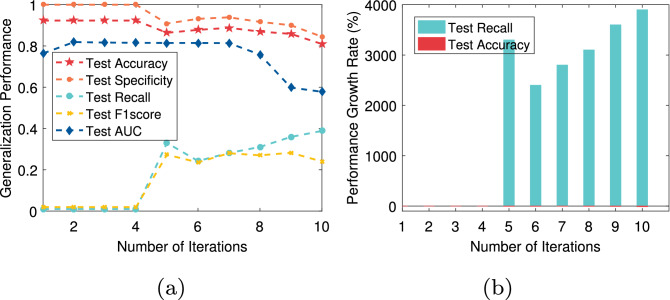
The test performance versus number of iteration on T2DM dataset: (**a**) generalization performance; (**b**) performance growth rate.

**Figure 6:**
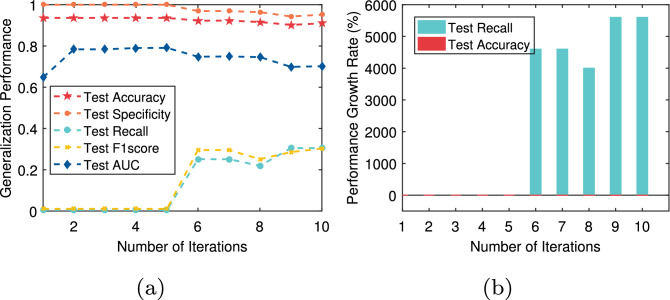
The test performance versus number of iteration on Pri_diab dataset: (**a**) generalization performance; (**b**) performance growth rate.

**Figure 7:**
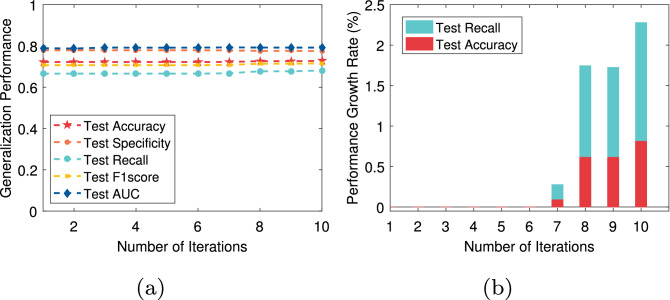
The test performance versus number of iteration on CVD dataset: (**a**) generalization performance; (**b**) performance growth rate.

In this paper, we not only pay attention to the overall accuracy of the model in the diagnosis of chronic diseases but also pay more attention to whether the model can accurately diagnose sick cases (positive samples). That is, we hope that the recall of the model is as high as possible on the premise that the overall accuracy is high. For T2DM, CVD, Fra_Heart, and Pri_diab datasets, we observe that the ratio of the number of correctly predicted sick cases to the total number of sick cases is low, that is, the recall rate is low. The reason is that there is a class imbalance problem in these datasets. To solve this problem, the AEPNN algorithm 2 is proposed in “Diagnostic framework for chronic diseases” section. Because the NLPNN algorithm is a strong classifier, we do not need too many individual classifiers, whose number is equal to the number of iterations. The test performance will change with the increase of the number of training rounds of the NLPNN algorithm. Although the overall diagnostic accuracy decreases slightly, the diagnostic accuracy of sick cases has been significantly improved. We choose the number of iterations corresponding to the maximum value of the difference between the growth rate of recall and the decrease rate of accuracy as the final number of training rounds of the NLPNN algorithm to obtain the best performance. Figures 4, 5, 6, 7 show the performance of the proposed algorithm when applied to the Fra_Heart, T2DM, Pri_diab, and CVD datasets at different iterations of NLPNN, respectively. Comprehensive analysis with Table 1, we can see that the higher the class imbalance ratio of chronic disease data, the more obvious AEPNN improves the recall.

The generalization performance of AEPNN on the Fra_Heart dataset is shown in Fig. 4a. The performance growth rate is calculated based on the number of NLPNN classifiers being one. From Fig. 4b, we observe that the recall has a growth rate of close to 300% when the number of NLPNN classifiers is six, which is chosen as the best number of NLPNN classifiers for the diagnosis of heart disease. The most surprising thing is the performance of AEPNN on the T2DM and Pri_diab datasets. As it can be seen from Figs. 5a and 6a, when the number of NLPNN is greater than four, the recall is significantly improved. When the number of NLPNN reaches ten, the growth rate of the recall approaches 4000% on the T2DM dataset and 6000% on the Pri_diab dataset. We can also know that the growth rate of recall is much higher than the decreased rate of accuracy from Figs. 5b and 6b.

From Fig. 7, we can see that although the performance of AEPNN on the CVD dataset is not significantly improved, the growth rate of recall is still higher than the decreased rate of accuracy. It indicates that the proposed algorithm is effective for the improvement of recall. The advantage it brings is that it can reduce the missed diagnosis rate for sick cases so that more patients with chronic diseases can treat and control the development of the disease in time. We also quantitatively compare the generalization performance of AEPNN and NLPNN algorithms by introducing $G\_mean = \sqrt{Recall * Specificity}$, which is a powerful indicator to evaluate the classification accuracy for class imbalanced datasets^49^. From Table 5, we can see that AEPNN can effectively improve $G\_mean$ by combining multiple NLPNNs. In particular, AEPNN can increase the $G\_mean$ from 0.0985 to 0.5728 on the T2DM dataset and from 0.0731 to 0.5385 on the Pri_diab dataset by combining ten NLPNNs.

**Table 5:** Performance of two proposed algorithms on four datasets with class imbalance.

Abbreviation		Acc	Re	F1	G_mean
T2DM	NLPNN	0.9232	0.0097	0.0192	0.0985
	AEPNN (10/10)	0.8095	0.3883	0.2402	0.5728
CVD	NLPNN	0.7265	0.6847	0.7161	0.7256
	AEPNN (10/10)	0.7279	0.6810	0.7160	0.7267
Fra_Heart	NLPNN	0.8726	0.0614	0.1148	0.2476
	AEPNN (6/10)	0.8219	0.2456	0.2705	0.4731
Pri_diab	NLPNN	0.9360	0.0053	0.0106	0.0731
	AEPNN (10/10)	0.9098	*0.3048	0.3032	0.5385

## Conclusion

In this paper, we have investigated a universal learning algorithm based on PNN for the early diagnosis of chronic diseases. Five state-of-the-art baseline algorithms are selected to compare with the NLPNN algorithm. Experiment results show that NLPNN achieves the best accuracy on the nine chronic disease datasets. In particular, for the early diagnosis of chronic kidney disease and breast cancer disease, the generalization accuracy, recall, specificity, and AUC value of this model have achieved 1.000, 1.000, 1.000, and 1.000, respectively. Furthermore, an AEPNN algorithm is further proposed to alleviate the class imbalance problem in chronic disease datasets. We aim to increase the probability of the sick cases being accurately diagnosed, that is, to increase the recall value of the model. Experiments on the four chronic disease datasets with class imbalance problems have confirmed the effectiveness of our model. It is noted that the AEPNN model performs best on the Pri_diab dataset with a positive-negative sample ratio of 1:12.78, and the growth rate of its recall is close to 6000%. The proposed algorithm can effectively assist chronic disease experts in quickly screening patients with chronic diseases, and save the cost of further testing for patients. It should be pointed out that although our algorithm performs better on small-scale datasets, the PNN-based model also shows great application potential on large-scale datasets, such as protein-protein interaction prediction and disease diagnosis based on medical images.

In future work, we will further investigate the PNN-based model in disease diagnosis. Although PNN can effectively capture hidden features parameter-free, there is still a problem with how to adaptively select the best-hidden features from the network architecture of PNN to achieve competitive performance. Thus, we consider combining PNN with computational intelligence algorithms (such as monarch butterfly optimization (MBO), earthworm optimization algorithm (EWA), and elephant herding optimization (EHO)) to improve the performance of disease diagnosis.

## Supplementary Information

Supplementary Information.

## Data Availability

The datasets used and/or analyzed during the current study available from the corresponding author on reasonable request.
