# Supplementary material for: A novel early diagnostic framework for chronic diseases with class imbalance
Source: Sci Rep. 2022 May 21;12:8614. doi: 10.1038/s41598-022-12574-x (PMC9123399; doi:10.1038/s41598-022-12574-x)
Supplement: Supplementary file 1 — Supplementary Information. [file 41598_2022_12574_MOESM1_ESM.pdf]

A Novel Early Diagnostic Framework for Chronic Diseases with Class Imbalance

Xiaohan Yuan, Shuyu Chen, Chuan Sun, Lu Yuwen

May 1, 2022

Appendix

1 Proof of Lemma 1

We first consider the partial derivative of the exponential loss function $\ell_{\exp}(h \mid \mathcal{D})$ with respect to $h(\boldsymbol{x})$:

$$\frac{\partial \ell_{\exp}(h \mid \mathcal{D})}{\partial h(\boldsymbol{x})} = -e^{-h(\boldsymbol{x})} P(f(\boldsymbol{x})=1 \mid \boldsymbol{x}) + e^{h(\boldsymbol{x})} P(f(\boldsymbol{x})=-1 \mid \boldsymbol{x}),$$

and set this equation equal to zero and get

$$h^*(\boldsymbol{x}) = \frac{1}{2} \ln \frac{P(f(\boldsymbol{x})=1 \mid \boldsymbol{x})}{P(f(\boldsymbol{x})=-1 \mid \boldsymbol{x})}.$$

In other words, the minimum value of the exponential loss function $\ell_{\exp}(h \mid \mathcal{D})$ can be obtained at $h^*(\boldsymbol{x})$. After ignoring $P(f(\boldsymbol{x})=1 \mid \boldsymbol{x}) > P(f(\boldsymbol{x})=-1 \mid \boldsymbol{x})$, the $h^*(\boldsymbol{x})$ satisfies

$$\begin{aligned}
\operatorname{sign}(h^*(\boldsymbol{x})) &= \operatorname{sign}\left(\frac{1}{2} \ln \frac{P(f(\boldsymbol{x})=1 \mid \boldsymbol{x})}{P(f(\boldsymbol{x})=-1 \mid \boldsymbol{x})}\right) \\
&= \begin{cases} 1, & P(f(\boldsymbol{x})=1 \mid \boldsymbol{x}) > P(f(\boldsymbol{x})=-1 \mid \boldsymbol{x}) \\ -1, & P(f(\boldsymbol{x})=1 \mid \boldsymbol{x}) < P(f(\boldsymbol{x})=-1 \mid \boldsymbol{x}) \end{cases} \\
&= \underset{y \in \{-1,1\}}{\arg\max} P(f(\boldsymbol{x})=y \mid \boldsymbol{x}),
\end{aligned}$$

which means that when the exponential loss function $\ell_{\exp}(h \,|\, \mathcal{D})$ is minimized, the classification error rate ϵ will also be minimized. This completes the proof.

2 Proof of Lemma 2

First, the relationship between the exponential loss function $\ell_{\exp}\left(\alpha_l h_l \mid \mathcal{D}_l\right)$ and the classification error ϵ_l can be written as follows

$$\begin{aligned}
&\ell_{\exp}\left(\alpha_l h_l \mid \mathcal{D}_l\right) = \int_{\boldsymbol{x}\sim\mathcal{D}_l} e^{-f(\boldsymbol{x})\alpha_l h_l(\boldsymbol{x})} p(\boldsymbol{x}) \mathrm{d}\boldsymbol{x} \\
&= \int_{\boldsymbol{x}\sim\mathcal{D}_l} [e^{-\alpha_1} \mathbb{I}\left(f(\boldsymbol{x}) = h_l(\boldsymbol{x})\right) + e^{\alpha_l}\left(f(\boldsymbol{x}) \neq h_l(\boldsymbol{x})\right)] p(\boldsymbol{x}) \mathrm{d}\boldsymbol{x} \\
&= e^{-\alpha_l} \int_{\boldsymbol{x}\sim\mathcal{D}_l} \mathbb{I}\left(f(\boldsymbol{x}) = h_l(\boldsymbol{x})\right) p(\boldsymbol{x}) \mathrm{d}\boldsymbol{x} + \\
&\quad e^{\alpha_l} \int_{\boldsymbol{x}\sim\mathcal{D}_l} \mathbb{I}\left(f(\boldsymbol{x}) \neq h_l(\boldsymbol{x})\right) p(\boldsymbol{x}) \mathrm{d}\boldsymbol{x} \\
&= e^{-\alpha_l}(1-\epsilon_l) + e^{\alpha_l}\epsilon_l,
\end{aligned}$$

and its derivative with respect to α_l is expressed as

$$\frac{\partial \ell_{\exp}\left(\alpha_l h_l \mid \mathcal{D}_l\right)}{\partial \alpha_l} = -e^{-\alpha_l}\left(1-\epsilon_l\right) + e^{\alpha_l}\epsilon_l.$$

Thus, the exponential loss function $\ell_{\exp}\left(\alpha_l h_l \mid \mathcal{D}_l\right)$ has the minimum value when

$$\alpha_l = \frac{1}{2}\ln\left(\frac{1-\epsilon_l}{\epsilon_l}\right).$$

This completes the proof.

3 Proof of Lemma 3

Obviously, the ideal classifier h_{l+1} satisfies

$$h_{l+1}(\boldsymbol{x}) = \arg\min_{h} \ \ell_{\exp}\left(H_l + h \mid \mathcal{D}\right),$$

and

$$\ell_{\exp}(H_l+h\,|\,\mathcal{D})=\int_{\boldsymbol{x}\sim\mathcal{D}}\left[e^{-f(\boldsymbol{x})(H_l(\boldsymbol{x})+h(\boldsymbol{x}))}\right]p(\boldsymbol{x})\mathrm{d}\boldsymbol{x}$$

$$=\int_{\boldsymbol{x}\sim\mathcal{D}}\left[e^{-f(\boldsymbol{x})H_l(\boldsymbol{x})}e^{-f(\boldsymbol{x})h(\boldsymbol{x})}\right]p(\boldsymbol{x})\mathrm{d}\boldsymbol{x}$$

$$\simeq\int_{\boldsymbol{x}\sim\mathcal{D}}[e^{-f(\boldsymbol{x})H_l(\boldsymbol{x})}(1-f(\boldsymbol{x})h(\boldsymbol{x})+\frac{f^2(\boldsymbol{x})h^2(\boldsymbol{x})}{2})]p(\boldsymbol{x})\mathrm{d}\boldsymbol{x}$$

$$=\int_{\boldsymbol{x}\sim\mathcal{D}}\left[e^{-f(\boldsymbol{x})H_l(\boldsymbol{x})}\left(1-f(\boldsymbol{x})h(\boldsymbol{x})+\frac{1}{2}\right)\right]p(\boldsymbol{x})\mathrm{d}\boldsymbol{x},$$

where $e^{f(\boldsymbol{x})h(\boldsymbol{x})}$ be approximately equal to its Taylor expansion. Thus, the next ideal classifier

$$h_{l+1}(\boldsymbol{x})=\arg\min_h\ \ell_{\exp}\left(H_l+h\mid\mathcal{D}\right)$$

$$=\arg\min_h\int_{\boldsymbol{x}\sim\mathcal{D}}\left[e^{-f(\boldsymbol{x})H_l(\boldsymbol{x})}\left(1-f(\boldsymbol{x})h(\boldsymbol{x})+\frac{1}{2}\right)\right]p(\boldsymbol{x})\mathrm{d}\boldsymbol{x}$$

$$=\arg\max_h\int_{\boldsymbol{x}\sim\mathcal{D}}\left[e^{-f(\boldsymbol{x})H_l(\boldsymbol{x})}f(\boldsymbol{x})h(\boldsymbol{x})\right]p(\boldsymbol{x})\mathrm{d}\boldsymbol{x}$$

$$=\arg\max_h\int_{\boldsymbol{x}\sim\mathcal{D}}[\frac{e^{-f(\boldsymbol{x})H_l(\boldsymbol{x})}}{\int_{\boldsymbol{x}\sim\mathcal{D}}[e^{-f(\boldsymbol{x})H_l(\boldsymbol{x})}]p(\boldsymbol{x})\mathrm{d}\boldsymbol{x}}f(\boldsymbol{x})h(\boldsymbol{x})]p(\boldsymbol{x})\mathrm{d}\boldsymbol{x},$$

where $\int_{\boldsymbol{x}\sim\mathcal{D}}\left[e^{-f(\boldsymbol{x})H_l(\boldsymbol{x})}\right]p(\boldsymbol{x})\mathrm{d}\boldsymbol{x}$ is a constant. Let $\mathcal{D}_{l+1}$ denote the distribution

$$\mathcal{D}_{l+1}(\boldsymbol{x})=\frac{\mathcal{D}(\boldsymbol{x})e^{-f(\boldsymbol{x})H_l(\boldsymbol{x})}}{\int_{\boldsymbol{x}\sim\mathcal{D}}[e^{-f(\boldsymbol{x})H_l(\boldsymbol{x})}]p(\boldsymbol{x})\mathrm{d}\boldsymbol{x}},$$

which is equivalent to let

$$h_{l+1}(\boldsymbol{x})=\arg\max_h\int_{\boldsymbol{x}\sim\mathcal{D}_{l+1}}\left[f(\boldsymbol{x})h(\boldsymbol{x})\right]p(\boldsymbol{x})\mathrm{d}\boldsymbol{x},$$

in the definition of expectation. Since $f(\boldsymbol{x}),h(\boldsymbol{x})\in\{-1,+1\}$, and

$$f(\boldsymbol{x})h(\boldsymbol{x})=1-2\mathbb{I}(f(\boldsymbol{x})\neq h(\boldsymbol{x})),$$

the ideal classifier h_{l+1} can eventually be expressed as

$$h_{l+1}(\boldsymbol{x})=\arg\min_h\int_{\boldsymbol{x}\sim\mathcal{D}_{l+1}}\mathbb{I}(f(\boldsymbol{x})\neq h(\boldsymbol{x}))p(\boldsymbol{x})\mathrm{d}\boldsymbol{x},$$

which will minimize the classification error under data distribution $\mathcal{D}_{l+1}$. Therefore, the classifier h_l should be trained based on the distribution $\mathcal{D}_l$, and the relationship between the distributions $\mathcal{D}_{l+1}$ and $\mathcal{D}_l$ is as follows

$$\begin{aligned}\mathcal{D}_{l+1}(\boldsymbol{x}) &= \frac{\mathcal{D}(\boldsymbol{x})e^{-f(\boldsymbol{x})H_l(\boldsymbol{x})}}{\int_{\boldsymbol{x}\sim\mathcal{D}}[e^{-f(\boldsymbol{x})H_l(\boldsymbol{x})}]p(\boldsymbol{x})\mathrm{d}\boldsymbol{x}} \\ &= \frac{\mathcal{D}(\boldsymbol{x})e^{-f(\boldsymbol{x})H_{l-1}(\boldsymbol{x})}e^{-f(\boldsymbol{x})\alpha_l h_l(\boldsymbol{x})}}{\int_{\boldsymbol{x}\sim\mathcal{D}}[e^{-f(\boldsymbol{x})H_l(\boldsymbol{x})}]p(\boldsymbol{x})\mathrm{d}\boldsymbol{x}} \\ &= \mathcal{D}_l(\boldsymbol{x})e^{-f(\boldsymbol{x})\alpha_l h_l(\boldsymbol{x})}\frac{\int_{\boldsymbol{x}\sim\mathcal{D}}e^{-f(\boldsymbol{x})H_{l-1}(\boldsymbol{x})}p(\boldsymbol{x})\mathrm{d}\boldsymbol{x}}{\int_{\boldsymbol{x}\sim\mathcal{D}}e^{-f(\boldsymbol{x})H_l(\boldsymbol{x})}p(\boldsymbol{x})\mathrm{d}\boldsymbol{x}}.\end{aligned}$$

This completes the proof.
